# Mitochondrial cytochrome *c* shot towards histone chaperone condensates in the nucleus

**DOI:** 10.1002/2211-5463.13176

**Published:** 2021-05-19

**Authors:** Katiuska González‐Arzola, Alejandra Guerra‐Castellano, Francisco Rivero‐Rodríguez, Miguel Á. Casado‐Combreras, Gonzalo Pérez‐Mejías, Antonio Díaz‐Quintana, Irene Díaz‐Moreno, Miguel A. De la Rosa

**Affiliations:** ^1^ Institute for Chemical Research (IIQ) Scientific Research Centre Isla de la Cartuja (cicCartuja) University of Seville – CSIC Spain

**Keywords:** chromatin remodelling, cytochrome *c*, DNA damage response, histone chaperone, liquid–liquid phase separation, lysine acetylation

## Abstract

Despite mitochondria being key for the control of cell homeostasis and fate, their role in DNA damage response is usually just regarded as an apoptotic trigger. However, growing evidence points to mitochondrial factors modulating nuclear functions. Remarkably, after DNA damage, cytochrome *c* (C*c*) interacts in the cell nucleus with a variety of well‐known histone chaperones, whose activity is competitively inhibited by the haem protein. As nuclear C*c* inhibits the nucleosome assembly/disassembly activity of histone chaperones, it might indeed affect chromatin dynamics and histone deposition on DNA. Several histone chaperones actually interact with C*c* Lys residues through their acidic regions, which are also involved in heterotypic interactions leading to liquid–liquid phase transitions responsible for the assembly of nuclear condensates, including heterochromatin. This relies on dynamic histone–DNA interactions that can be modulated by acetylation of specific histone Lys residues. Thus, C*c* may have a major regulatory role in DNA repair by fine‐tuning nucleosome assembly activity and likely nuclear condensate formation.

## Graphical abstract

DNA damage induces liquid–liquid phase separation within DNA foci, thereby allowing repair mechanisms to access damaged sites. DNA damage also makes mitochondrial cytochrome *c* translocate to the nucleus to sequester histone chaperones, for example SET/TAF‐Iβ, and impair their functions. We thus hypothesize that the INHAT activity in chromatin remodelling might be inhibited, as SET/TAF‐Iβ is a component of the INHAT complex.

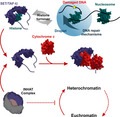

**Abbreviations**
AIF: apoptosis inducing factor
ANP32: acidic leucine‐rich nuclear phosphoprotein 32 family
ANP32A: acidic leucine‐rich nuclear phosphoprotein 32 family member A
ANP32B: acidic leucine‐rich nuclear phosphoprotein 32 family member B
ANP32E: acidic leucine‐rich nuclear phosphoprotein 32 family member E
Apaf‐1: apoptosis protease‐activating factor‐1
APLF: aprataxin‐PNK‐like factor
ASF1: anti‐silencing function 1
ATM: ataxia telangiectasia modified
ATR: ataxia telangiectasia and Rad3 related
BAK: Bcl‐2 homologous antagonist/killer
BAX: Bcl‐2‐associated X protein
bZLM: basic leucine zipper‐like motif
CAF‐1: chromatin assembly factor 1
CBP: CREB‐binding protein
C*c*
: cytochrome *c*
DAXX: death domain‐associated protein
DDR: DNA damage response
DDX3X: DEAD box RNA helicase 3, X‐linked
Ddx4: DEAD‐box helicase 4
DNA‐PKcs: DNA‐dependent protein kinase catalytic subunits
DSB: double‐strand break
EWS: ewing sarcoma
FACT: facilitates chromatin transcription
FoxO1: forkhead box protein O1
FUS/TLS: fused in sarcoma/translocated in sarcoma
HAT: histone acetyltransferase
HDAC: histone deacetylase
HIRA: histone regulator A
hnRNP A2: heterogeneous nuclear ribonucleoprotein A2
hnRNP C1/C2: heterogeneous nuclear ribonucleoproteins C1 and C2
hnRNP: heterogeneous nuclear ribonucleoprotein
HP1: heterochromatin protein 1
IDR: intrinsically disordered region
INHAT: inhibitor of acetyltransferases
Ino80: inositol‐requiring 80
IP3R: inositol 1,4,5‐triphosphate receptor
KAP1: KRAB‐associated corepressor
LC: low complexity
LCAR: low complexity acidic region
LCD: low‐complexity domain
LLPS: liquid–liquid phase separation
LRR: leucine‐rich region
NAP: nucleosome assembly protein
NAP1L1/NAP1L4: nucleosome assembly protein 1‐like 1/4
NCL: nucleolin
NHEJ: non‐homologous end joining
NLS: nuclear localization signal
NMR: nuclear magnetic resonance
NPM: nucleophosmin
NRP1: nucleosome assembly protein 1 (NAP1)‐related protein 1
PARP1: poly(ADP‐ribose) polymerase 1
P‐bodies: processing bodies
PCAF: p300/CBP‐associated factor
PP2A: protein phosphatase 2A
pp32: phosphoprotein of 32 kDa
PRMT1: protein‐arginine methyl transferase 1
PTM: post‐translational modification
PUMA: p53‐upregulated modulator of apoptosis
RBP: RNA‐binding protein
RGG: arginine–glycine–glycine
RNP: ribonuclear protein
RRM: RNA recognition motif
SET/TAF‐Iβ: SET/template‐activating factor‐Iβ
TAF15: TATA box‐binding protein‐associated factor 68 kDa
TAF‐Iα: template‐activating factor‐Iα
U‐bodies: uridine‐rich small nuclear RNA bodies
XLF: XRCC4‐like factor
XRCC4: X‐ray repair cross‐complementing protein 4

Mitochondria play an essential role in cell metabolism and take part in the core of cell signalling networks that sense and coordinate responses to environmental changes. The control of mitochondrial state—via mitophagy, translation attenuation, unfolded protein response activated by mistargeting, mitochondrial unfolded protein response or mDNA damage regulation [1, 2]—involves extra‐mitochondrial factors and expression of nuclear genes. In fact, some of them are induced by mitochondrial transcription factors, for example during the retrograde response. Moreover, a set of mitochondrial factors, either encoded in the mitochondria, such as the mitochondria‐derived peptides [3], or in the nucleus, such as the apoptosis‐inducing factor (AIF) and cytochrome *c* (C*c*), are key players in cell fate decisions [4, 5].

Mitochondria are also end‐targets of apoptosis signalling elicited by strong nuclear DNA damage. The DNA damage response (DDR), mediated by p53, eventually activates proteins such as PUMA (p53 upregulated modulator of apoptosis), BAX (Bcl‐2‐associated X protein) and BAK (Bcl‐2 homologous antagonist/killer), thereby yielding the release of pro‐apoptotic factors from mitochondria [6]. Notably, one of the three known DDR early sensors, the ataxia telangiectasia and Rad3‐related (ATR) protein, plays a dual role depending on its isomerization state: one state aids the onset of DDR upstream of p53 in the cell nucleus and the other state plays a protective role against pro‐apoptotic stimuli in mitochondria [7]. This illustrates how mitochondrial reactions can be modulated during the DDR response, but does not imply a direct involvement of mitochondrial proteins in regulation of nuclear DNA repair or DDR.

It was once assumed that the biological function of C*c* was confined to mitochondria and restricted to its ability to connect complexes III and IV in the electron transport chain. The functionality of C*c* is indeed controlled *in vivo* by several post‐translational modifications (PTMs) [8, 9, 10, 11, 12, 13, 14, 15, 16, 17]. Such a canonical function of the haem protein was however questioned with the discovery that C*c* is released from mitochondria to cytosol upon treatment of cells with the apoptotic inducer staurosporine [5]. Afterwards, the apoptotic ability of the haem protein translocated into the cytosol was extended to other genotoxic treatments, such as etoposide, ultraviolet irradiation, actinomycin D or H_2_O_2_‐mediated oxidative stress [18, 19]. In the cytosol, C*c* interacts with the apoptosis‐activating factor 1 (Apaf‐1), triggering (a) apoptosome assembly, (b) the subsequent activation of downstream caspases and (c) controlled cell dismantlement [20, 21, 22]. C*c* also binds to the inositol 1,4,5‐triphosphate receptor (IP3R) at the endoplasmic reticulum membrane. This further stimulates massive C*c* release and, consequently, apoptosis [23, 24]. In fact, the sequence of events reaches a critical point of ‘no return’ in the execution of apoptosis [5]. Oxidation of the lipid cardiolipin by C*c* at the onset of apoptosis is indeed a decisive step [25].

Beyond cytosolic C*c* being a key element in apoptosis, several findings have led to the emergence of C*c* as a pleiotropic mitochondrial factor that migrates to the cell nucleus upon DNA damage both in mammals and plants [26, 27, 28, 29, 30]. Mitochondrial C*c* in the nucleus targets histone chaperones that might share common structural—and probably functional—features (Fig. 1). In this review, we summarize major aspects of C*c* signalling in the cell nucleus, describe the structure‐to‐function relationships of reported nuclear targets and discuss the biological consequences of various interactions.

**Fig. 1:**
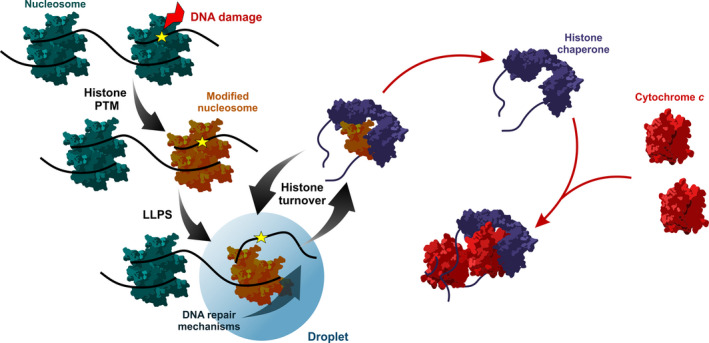
Role of histone chaperones in DNA damage‐induced LLPS and proposed regulation by C*c*. The DDR induces histone PTMs, for example acetylation and ubiquitination, and subsequent recruitment of DNA foci constituents. DNA repair foci are condensates formed upon LLPS events yielding droplets, which provides a unique environment for DNA repair. Histone chaperones contribute by mediating histone eviction and deposition, thus allowing components of DNA repair mechanisms to access the damaged site. In addition, mitochondrial C*c* migrates to the cell nucleus to interact with histone chaperones by their acidic regions in a manner similar to histones. In this way, C*c* may contribute to the regulation of DNA repair by fine‐tuning histone turnover.

## Mitochondrial cytochrome *c* as a signalling factor of DNA lesions in the nucleus

In addition to the consideration of cytosolic C*c* as a key element in apoptosis, an exciting discovery has been the observation of this metalloprotein migrating to the cell nucleus following DNA damage. New putative functions for nuclear C*c* are thus now emerging. Redistribution of C*c* and Apaf‐1 to the nucleus during apoptosis induced by actinomycin D—a drug that generates DNA breaks—was first reported by Ruíz‐Vela and coworkers [29]. Later, Nur‐E‐Kamal *et al*. [30] stated that C*c* gradually accumulates in the nucleus of HeLa cells upon applying the DNA‐damage inducer camptothecin. Remarkably, nuclear C*c* accumulation correlates with nuclear pyknosis during apoptosis, thereby contributing to chromatin remodelling and condensation [30].

Another notable finding revealed that C*c* migrates to the cell nucleus soon after drug‐induced DNA damage, even before triggering the caspase cascade and apoptosome formation in the cytosol [31]. Later, C*c* was found to be diffuse and faintly located in the cytosol, but abundantly distributed in the nuclei of HeLa cells upon treatment with actinomycin D [32]. Again, the haem protein was detectable in the cell nucleus prior to caspase cascade activation or apoptosis induction [32]. Recently, nuclear translocation of C*c* induced by copper has been found in neuroblastoma cells [33]. Such outcomes hint to novel functions for nuclear C*c*, beyond the well‐known roles in the cytosol and mitochondria.

Several proteomic analyses by our group served to identify an ample set of proteins that bind to extra‐mitochondrial C*c* following DNA breaks in humans and plants [26, 27, 28]. Such new interactions constitute a complex C*c*‐centred cell death signalling network. In fact, the haem protein plays a dual role in leading living cells to death not only by inhibiting pro‐survival routes but also by triggering pro‐apoptotic pathways [26, 27, 28, 34, 35]. Upon induction of DNA breaks, C*c* binds to a series of chromatin‐binding factors in the nuclei of both human and plant cells [26, 27]. These findings highlight the multi‐functional role of C*c* during the onset of apoptosis triggered by DNA breaks and suggest a previously unsuspected role for the hemeprotein in chromatin remodelling for DNA damage repair.

## Role of chromatin modifiers in DNA foci and their regulation by nuclear cytochrome *c*

A plethora of endogenous and exogenous sources cause different types of DNA damage, with double‐strand breaks (DSBs) being among the most toxic type of lesions as they can lead to chromosomal translocations and cancer development [36, 37]. Cells respond to DSBs by activating a pathway, the so‐called DDR, which is a well‐orchestrated network of cellular routes, including initial recognition, signal amplification, activation of cell cycle checkpoints and repair of DNA lesions [38, 39].

Within seconds to minutes following any DNA break, repair and checkpoint proteins are recruited to DSB sites, leading to the formation of DNA repair foci [40, 41, 42]. These foci are massive in comparison with the small size of a DSB itself [43]. The massive accumulation of DNA repair factors at foci apparently rapidly magnifies signalling, in such a way that a single break is sufficient to induce a large response and to arrest the cell cycle [44, 45]. Such rapid signal amplification is essential for preserving the genome and preventing cells with DSBs from entering mitosis [43].

DNA repair foci were initially observed in mammalian cells with the DNA repair protein Rad51 [46] and later with additional proteins that respond to DSBs [47, 48]. It was later observed that, immediately after DSB induction, ataxia telangiectasia modified (ATM) and other kinases phosphorylate C‐terminal Ser residues (Ser136 and Ser139) of histone H2AX—a variant of H2A—at the DSB site [40, 49]. Phosphorylated H2AX (γH2AX) is detectable within minutes after DNA break and aids the recruitment of other DDR proteins to DSB sites [50, 51, 52]. The number of foci formed during DDR is routinely used to assess the intensity of DNA damage and repair kinetics [53, 54, 55].

Recent studies demonstrate that DNA repair proteins assemble as DNA repair foci via liquid–liquid phase separation (LLPS) [56]. DNA repair foci form clusters by fusing with one another over time in mammalian cells [57, 58] and yeasts [59, 60]. Similar cellular condensates—also known as membraneless compartments—can be found in different cellular locations. For example, stress granules, processing bodies (P‐bodies), uridine‐rich small nuclear RNA bodies (U‐bodies) and centrosomes can be detected in the cytoplasm [56, 61, 62, 63]. In contrast, nucleoli, DNA repair foci, Cajal bodies, heterochromatin, nuclear speckles and histone locus bodies are found in the nucleus [64, 65, 66]. Some of them are however ubiquitous, as Cajal bodies, nucleoli or P‐bodies. Other condensates (e.g. DNA repair foci, stress granules or paraspeckles) appear after certain stimuli in specific cell types [56]. The main forces driving LLPS are multivalent weak interactions involving signalling domains repetitively included in RNA/DNA and/or proteins. The latter often contain intrinsically disordered, low complexity (LC) domains that can be regulated by PTMs [67].

In yeast, DNA repair foci are assembled through the fusion of liquid‐like bodies of Rad52 protein surrounding different DSBs within the nucleus [68]. Truncation of Rad52 intrinsically disordered region (IDR) avoids phase separation and increases cell sensitivity to DNA damage, highlighting the role of Rad52‐mediated phase transitions in the DNA repair process [68]. Upon DNA damage, poly(ADP‐ribose) polymerase 1 (PARP1) localizes to DNA damage sites and its auto‐poly(ADP‐ribosyl)ation triggers the recruitment of several proteins, for example fused in sarcoma/translocated in sarcoma (FUS/TLS), ewing sarcoma (EWS) and TATA box‐binding protein‐associated factor 68 kDa (TAF15), also abbreviated as FET proteins [69]. PARP1‐mediated clustering of FET proteins around the DSB causes phase separation, which leads to DNA repair foci formation [69]. The exact role of these condensates in the DDR is unknown, but they improve DNA repair efficiency somehow [56]. Phase separation and DNA foci formation involve multivalent weak interactions between poly(ADP‐ribose) and the arginine–glycine–glycine (RGG) domain, along with the LCDs of FET proteins [56, 69]. Consequently, preventing the assembly of DNA repair foci via PARP1 inhibition leads to neurodegenerative diseases [70].

In addition to liquid‐like DNA repair foci formation, DDR affects the overall chromatin structure to enable the access of repair proteins to the DNA injury site. DSB repair requires profound chromatin rearrangements to sense damage and to aid the approach of repair machinery [71]. During DNA damage sensing and repair, histones undergo PTMs, including phosphorylation, acetylation, methylation and ubiquitination. Such modifications act as beacons for recruiting proteins involved in DDR [72].

Chromatin restoration at repair sites involves the deposition of newly synthesized histones, as shown for histone variants H2A, H3.1 and H3.3 [73, 74, 75, 76, 77]. New histone laying and chromatin reshaping following DSBs requires dedicated histone chaperones [78], including histone regulator A (HIRA) [74], chromatin assembly factor 1 (CAF‐1) [73], facilitates chromatin transcription (FACT) [75], nucleolin (NCL) [79], aprataxin‐PNK‐like factor (APLF) [80], anti‐silencing function 1 (ASF1) [81], death domain‐associated protein (DAXX) [82], p400 remodelling ATPase [83], inositol‐requiring 80 (Ino80) [84], nucleosome assembly protein 1‐like 1 and 4 (NAP1L1 and NAP1L4) [85], acidic nuclear phosphoprotein 32 family member E (ANP32E) [86] and SET/template‐activating factor (TAF)‐Iβ (SET/TAF‐Iβ) [87].

Notably, nuclear C*c* binds various histone chaperones following DNA breaks, suggesting the hemeprotein assists DNA repair regulation. In the following subsections, we review and discuss the main findings regarding regulation of the DDR by some histone chaperones and C*c* in the context of genotoxic stress.

### SET/template‐activating factor‐Iβ

SET/TAF‐Iβ is a protein involved in a wide variety of biological processes, namely cell cycle control [88], replication [88, 89, 90], transcription and chromatin remodelling [91], and apoptosis [92]. SET/TAF‐Iβ was first described as a translocated gene in acute undifferentiated leukaemia [93] and was found to be upregulated in diverse kinds of tumours [94, 95]. For this reason, it has been considered as an oncoprotein. SET/TAF‐Iβ forms a dimer that assumes a headphone‐like shape. Each monomer consists of an N‐terminal backbone helix involved in dimerization, an earmuff domain and a long acidic stretch in the C‐end [96].

Within the context of the DDR, SET/TAF‐Iβ has been described to be involved in the regulation of this process at several stages, as addressed hereafter. First, it is well established that SET/TAF‐Iβ acts as a histone chaperone of the nucleosome assembly protein (NAP) family, whose members are capable of disassembling nucleosomes in an ATP‐independent manner [96, 97]. This explains the crucial role of histone chaperones during the DDR of facilitating the entry of DNA repair proteins into the damaged site [98, 99, 100, 101] and allowing chromatin dismantling, repair and rearrangement in a quick and precise manner [99, 102]. During this process, histone chaperones meet the demand for histone supplies and promote proper nucleosome assembly and the recycling of modified histones evicted from chromatin [103, 104]. Therefore, SET/TAF‐Iβ is considered to be an important factor in chromatin dynamics and remodelling, with special emphasis on its influence on DNA repair [105, 106]. In fact, the histone chaperone activity of SET/TAF‐Iβ is crucial for the regulation of cell survival upon exposure to DNA‐damaging agents [87].

Furthermore, SET/TAF‐Iβ is a key subunit of the inhibitor of acetyltransferases complex, or INHAT [105]. Acetylation and deacetylation are mediated by families of histone acetyltransferases (HATs) and histone deacetylases (HDACs), respectively [107, 108]. The INHAT complex usually comprises two other proteins, namely template‐activating factor‐Iα (TAF‐Iα) and acidic Leu‐rich nuclear phosphoprotein 32 family member A (ANP32A, a.k.a. phosphoprotein of 32 kDa or pp32). This large complex exerts a negative regulatory effect over the HAT activity of p300, the CREB‐binding protein (CBP) and the p300/CBP‐associated factor (PCAF) [105, 109, 110, 111].

It has also been reported that SET/TAF‐Iβ inhibits the p300/CBP‐ and PCAF‐mediated acetylation of non‐histone proteins, namely the tumour suppressor p53 [112], the forkhead box protein O1 (FoxO1) [113] and the well‐known DDR player Ku70 [114]. The Ku70/Ku80 heterodimer binds to the DNA ends of DSBs as a first step of the non‐homologous end‐joining (NHEJ) DNA repair pathway. Then, Ku70/Ku80 recruits other NHEJ effectors, including DNA‐dependent protein kinase catalytic subunits (DNA‐PKcs), X‐ray repair cross‐complementing protein 4 (XRCC4), ligase IV, XRCC4‐like factor (XLF) or the nuclease Artemis [115], thus allowing the repair process. Interestingly, it has recently been reported that Ku70/Ku80 is bound to SET/TAF‐Iβ through the C‐terminal end of the histone chaperone in the homeostatic cell nucleus, impeding the binding of the former to non‐damaged DNA [114]. However, upon DNA damage, the complex dissociates and releases Ku70/Ku80, which is then capable of binding to DSBs and initiating the NHEJ pathway. Thus, SET/TAF‐Iβ physiologically downregulates NHEJ‐mediated DNA repair and, hence, the DDR [114]. Concomitantly, CBP and PCAF can acetylate several Lys residues of Ku70, causing the release of Bax from the Ku70‐Bax complex and triggering Bax‐mediated apoptosis [116]. This process is also inhibited by the INHAT activity of SET/TAF‐Iβ [114], conferring its status as an oncoprotein. Of note, SET/TAF‐Iβ also finetunes cell survival and proliferation by exerting its INHAT activity over the tumour suppressor p53 [112, 117] and the transcription factor FoxO1 [113].

Recent studies have revealed how SET/TAF‐Iβ modulates the DDR by directly acting on the DNA foci. When a DSB occurs, signal transducer kinases are recruited by DSB sensor proteins and activate several DDR mechanisms. Among them, ATM kinase phosphorylates the KRAB‐associated corepressor (KAP1) [118], which subsequently phosphorylates heterochromatin protein 1 (HP1). HP1 phosphorylation triggers its release from chromatin together with CHD3, which is a fundamental pre‐requisite for chromatin relaxation, as well as to allow DNA repair mechanisms to access DNA lesions [98, 119, 120]. A model has recently been proposed in which SET/TAF‐Iβ interacts with KAP1 upon DNA damage and retains it bound to chromatin. Therefore, chromatin resection and relaxation are impaired, and DNA repair processes slow down [121].

Last but not least, SET/TAF‐Iβ is a well‐known inhibitor of protein phosphatase 2A (PP2A). PP2A is one of the main serine–threonine protein phosphatases in mammalian cells [122, 123] that regulates a wide variety of cellular processes, namely the cell cycle, metabolism, DNA replication, transcription and translation, cell proliferation and apoptosis [124, 125, 126, 127]. Moreover, PP2A regulates the DDR at several levels by controlling the phosphorylation state of DDR signal factors. Indeed, PP2A dephosphorylates the DDR transducer kinase ATM and DNA‐PK, as well as Ku70/Ku80 accessory subunits, thus diminishing their activity and promoting the repair of injured DNA [128, 129]. In light of the above, PP2A inhibition by SET/TAF‐Iβ reflects another level at which the histone chaperone regulates diverse steps of the DDR.

Interestingly, when mitochondrial C*c* reaches the nucleus upon DNA damage, the hemeprotein binds to SET/TAF‐Iβ and competes with histones for binding to the chaperone [31]. This results in inhibition of the nucleosome assembly/disassembly activity of SET/TAF‐Iβ, directly affecting its function on chromatin dynamics [31]. This process may slow down histone deposition/eviction on damaged DNA by SET/TAF‐Iβ. Since C*c* and histones compete with each other for binding to SET/TAF‐Iβ with similar affinity constants, a sufficient C*c* concentration in the nucleus would shift histones out of the complex with the histone chaperone [130]. Furthermore, the C*c*:SET/TAF‐Iβ interaction could have additional effects on DDR‐related functions described for SET/TAF‐Iβ, expanding the regulatory role of nuclear C*c*. Given its histone chaperone activity, SET/TAF‐Iβ largely contributes to structural chromatin remodelling [91]. It is thus tempting to hypothetize that the interaction of SET/TAF‐Iβ with C*c* might interfere in its role as a gene transcription activator, thereby resulting in transcription repression.

### Nucleosome assembly protein 1‐related protein 1

The nucleosome assembly protein 1 (NAP1)‐related protein 1 (NRP1) belongs to the NAP1 family of histone chaperones. NRP1 is the plant orthologue of SET/TAF‐Iβ, sharing a high degree of structural homology [131]. A homology model of NRP1 resembling the structure of human SET/TAF‐Iβ showed a headphone‐shaped homodimer composed of a long backbone helix at its N‐terminal region responsible for dimerization and a C‐terminal earmuff domain which likely acts as a histone chaperone [132]. In addition, it has been posited that DDR mechanisms are highly conserved in plants with respect to other eukaryotic organisms [133]. These findings invite the possibility that functions are shared between NRP1 and its human counterpart SET/TAF‐Iβ. Much like SET/TAF‐Iβ, NRP1 regulates replication, transcription and cellular division during plant growth and development as well as DNA repair [134, 135, 136, 137, 138] due to its ability to assemble and disassemble nucleosomes [132, 139].

Like any other histone chaperone, NRP1 binds to both H2A‐H2B [137, 140] and H3‐H4 histone dimers [132]. As mentioned above, NRP1 participates in transient chromatin assembly and disassembly events [85, 132]. These processes are crucial for homologous recombination repair, which is essential for genome integrity in plants [140]. Several works have suggested an important role for NRP1 in genomic integrity maintenance [140, 141]. Specifically, NRP1 gathers in plant cell nuclei upon DSB induction, suggesting its role in the plant DDR. Thus, NRP1 modulates chromatin dynamics, which influences the ability of DNA repair effectors to accomplish their function [132]. It has been proposed that not only does NRP1 promote homologous recombination synergistically with ATP‐dependent chromatin‐remodelling factor Ino80, but also that this mechanism is triggered by the formation of γH2AX foci [142]. Additionally, NRP1 causes a decrease in the content of the H2A.Z histone variant in nucleosomes under standard growing conditions [143].

Much like SET/TAF‐Iβ, NRP1 accumulates in the cell nucleus upon DNA damage and inhibits plant PP2A [144, 145]. The functional consequences of such interactions have not yet been elucidated. However, it is tempting to propose that NRP1‐mediated PP2A inhibition has similar consequences in plants and mammals.

As mentioned above, plant C*c* reaches the cell nucleus, where it interacts with NRP1, upon DNA damage stimuli. Such an interaction impairs the histone chaperone activity of NRP1, thereby suggesting that C*c* modulates the DDR in a concentration‐dependent manner [130, 132]. Like human SET/TAF‐Iβ, plant NRP1 regulates gene transcription due to its ability to assemble/disassemble nucleosomes [134, 135, 136, 137, 138]; therefore, the interaction of NRP1 with C*c* in plants might negatively affect the transcription of genes involved in growth, development and/or DNA repair. The presence of similar mechanisms in both mammalian and plant cells suggests that they are largely conserved throughout evolution.

### Acidic Leu‐rich nuclear phosphoprotein 32 family member B

The members of the ANP32 family stand out from other histone chaperone groups because of their divergent roles within the cell [146]. For instance, mammalian ANP32 proteins have been reported to participate in death regulatory pathways. Within this context, several studies have shown that ANP32 proteins aid in apoptosome formation by stabilizing Apaf‐1 [147, 148]. Moreover, the ANP32 family member A (ANP32A) directly promotes caspase‐3 activation [149]. In contrast, the ANP32 family member B (ANP32B), which shares 81% sequence homology with ANP32A, has been described as a caspase‐3 substrate and inhibitor, suggesting antagonistic regulatory roles for ANP32A and ANP32B during cell death [146, 150, 151]. ANP32B, like other members of its family, displays a structured N‐terminal domain with four Leu‐rich regions (LRRs), and a C‐terminal low complexity acidic region (LCAR) composed of negatively charged residues [152].

As histone chaperones, ANP32 family members participate in transcriptional regulation and configuration of chromatin architecture [146, 152]. Diverse studies have shown that both ANP32A and ANP32B modulate transcription by facilitating nucleosome rearrangement around the promoters of specific genes [153, 154, 155, 156, 157]. This activity must be guided by transcriptional factors, for example Krüpper‐like transcription factor 5 [155, 158]. Nucleosome assembly assays showed that ANP32B histone chaperone activity relies on its N‐terminal structured domain [158]. In fact, the N‐end domain specifically binds to the H3‐H4 histone dimer, whereas the C‐end LCAR binds to the H2A‐H2B dimer, thus increasing ANP32B affinity towards the nucleosome and facilitating its nucleosome assembly activity [158].

The role of ANP32B during the DDR is not fully elucidated, although it is known to bind to C*c* upon DNA damage [27]. The hemeprotein could thus regulate the histone chaperone activity of ANP32B, as already described for SET/TAF‐Iβ and NRP1 [28, 31, 132, 159]. Within this context, C*c* in the nucleus acquires a major regulatory role in the DNA repair process by fine‐tuning the nucleosome assembly activity of histone chaperones.

Other members of the ANP32 protein family also act as histone binding proteins. ANP32A participates in the INHAT complex [105] and, in particular, inhibits histone PTMs by binding unmodified histone H3 tails [160, 161]. In turn, the ANP32 family member E (ANP32E) binds specifically to the histone variant H2A.Z while associated with the p400/Tip60 complex [155, 162]. Notably, the ANP32E LCAR comprises a precise sequence—absent in other ANP32 family members like ANP32A or ANP32B—that yields binding specificity towards H2A.Z [155, 162].

### Nucleolin

Nucleolin (NCL) likewise interacts with C*c* in the cell nucleus following DNA damage [27]. NCL is a multifunctional phosphoprotein localized mainly in the nucleolus, being one of the most abundant non‐ribosomal proteins of such membrane‐less organelles [163, 164]. NCL also transits to the nucleoplasm in response to genotoxic stress. Like any RNA‐binding protein (RBP), NCL is involved in several aspects of DNA metabolism, participating broadly in DNA/RNA regulation, for example transcription, ribosome assembly or mRNA stability and translation [165, 166]. Several reports suggest that NCL promotes cell proliferation, since its amount closely correlates with the proliferative status of cells. As NCL is upregulated in tumours, it is widely used as a marker of cell proliferation [167, 168, 169]. Furthermore, NCL participates directly in the cellular response to DNA damage elicited upon UV and ionizing radiation [170, 171].

Remarkably, NCL interacts with γ‐H2AX followed by its recruitment around the DSB foci induced by camptothecin treatment [79]. It is also involved in the activation of ATM kinase and the formation of Rad51 foci following UV or camptothecin exposure [172]. NCL is composed of three main domains: an N‐terminal domain‐containing several Asp/Glu‐rich acidic stretches, a central domain comprising four RNA recognition motifs (RRM) and a C‐terminal domain rich in RGG repeats. The exact contribution of the N‐terminal domain for NCL function is unknown, but it contains numerous phosphorylation sites which are essential for NCL function [169]. The acidic stretches at the N‐terminal region have been proposed to bind histone H1 to induce chromatin decondensation [165, 173]. The central domain has been the focus of several structural studies, showing that this stretch of RRMs specifically recognizes RNA [169, 174, 175]. The C‐terminal domain contains RGG repeats interspersed with other amino acids, usually aromatic in nature. The RGG region is responsible for non‐specific interactions with nucleic acids that, however, facilitate the specific binding of the central RRM platform to RNA [176, 177]. These regions have also been described as protein–protein interaction domains since they recognize several core ribosomal proteins [178, 179].

Nucleolin possesses histone chaperone activity, which greatly enhances the action of the chromatin remodelling machinery [180]. Thus, NCL promotes the destabilization of the histone octamer, allowing the dissociation of H2A‐H2B dimers to facilitate chromatin transcription [180, 181]. NCL is recruited to sites of DNA breaks via binding to DNA repair protein RAD50, and it removes histones H2A and H2B from the nucleosome at the break site [182]. Such NCL‐dependent nucleosome disruption is necessary both for gathering DSB repair factors and for efficient DNA repair [182]. Interestingly, recruitment of NCL to the DSB results from the interaction of its RGG domain with the RAD50 protein [182]. Implications for the DDR or gene transcription repression of the interaction between NCL and C*c* in the nucleus following DNA breaks have not been explored yet.

### Heterogeneous nuclear ribonucleoprotein C1 and C2

Heterogeneous nuclear ribonucleoproteins (hnRNPs) form a significant subclass of known ribonuclear proteins (RNPs). These proteins escort RNA from transcription in the nucleus to translation in the cytoplasm. Accordingly, hnRNPs are responsible for packaging, processing and exporting of pre‐mRNA molecules [183, 184]. They are also involved in gene regulation through a variety of protein–protein, protein–RNA and protein–DNA interactions [184]. hnRNP C1 and hnRNP C2 are splice variants which differ by a 13‐amino acid stretch present in the middle of the coding sequence of the C2 gene [184, 185] and are frequently referred to as hnRNP C1/C2 or simply hnRNP C [184]. Specifically, hnRNP C1/C2 proteins have been shown to be involved in mRNA transcript packaging, splicing, nuclear retention and mRNA stability [183]. Under normal conditions, they are both located in the nucleoplasm, but not in nucleoli [186]. hnRNP C1/C2 proteins associate with RNA as tetramers formed by three hnRNP C1 subunits and one hnRNP C2 subunit, with an arrangement that seems to be critical for nucleic acid‐binding [187]. Each monomer contains a single RRM, a delineated nuclear localization signal (NLS), a basic leucine zipper‐like motif (bZLM) and an acidic auxiliary domain [187].

hnRNP C1/C2 are also nucleosome remodelling proteins that bind chromatin in response to genomic damage [184, 186]. Experiments analysing general stress response pathways suggest a role for these proteins in the DDR [184]. Despite their DNA damage‐induced chromatin‐binding ability, hnRNP C1/C2 are not actively recruited to the sites of DNA breaks [186]. Consequently, they might be involved in the functioning of chromatin in a global context, rather than in specifically targeting DNA breaks [186]. It has been proposed that hnRNP C1/C2 may play an indirect role in the DDR by coordinating the changes in gene expression required for DNA repair after irradiation through direct interaction with genomic DNA, DNA‐associated proteins and/or mRNA transcripts [184, 186]. The hnRNP C1/C2 proteins bind to the Ku protein complexed to RNA transcripts and can be phosphorylated by the catalytic subunit of the DNA‐dependent protein kinase [188]. This suggests a possible role for hnRNP C1/C2 in DNA DSB repair through the NHEJ pathway [186]. Other studies have connected hnRNP C1/C2 with telomere repair and maintenance [189]. Similarly to the above‐described NCL:C*c* complex, the DNA damage implications of the hnRNP C1/C2:C*c* complexes are not fully understood yet.

## Acidic regions as main targets for cytochrome *c*

Since the mid‐1980s, it has been known that non‐histone chromosomal proteins are enriched with certain regions primarily composed of acidic amino acids [190]. Such acidic tails could indeed play an important role in anchoring proteins to basic histones [191] and regulating nucleosome assembly and disassembly [192, 193, 194]. The role of histone chaperones in chromatin reorganization has been widely studied, with particular focus on the role of their acidic regions [195, 196].

From a structural point of view, histone chaperones exhibit a wide variety of different motifs, but a common feature is the presence of acidic stretches with a high content of glutamates and aspartates. These domains are often found near the C‐terminal end of histone chaperones and are usually disordered in the absence of any partner [197]. At physiological pH, histones are positively charged, and hence, their interaction with DNA is electrostatically driven. However, their positive charges allow them to engage in undesirable interactions with diverse acidic components of the cell, which may result in protein aggregates [196]. The acidic regions of histone chaperones thus enable them not only to bind histones to prevent their aggregation, but also to escort histones throughout their synthesis, transport and assembly/disassembly from DNA molecules [195, 197, 198].

Acidic disordered stretches, a.k.a. LCARs, become effective ‘readers’ of positively charged histones through electrostatic interactions. Such molecular recognition is improved by additional contacts between the folded regions of histone chaperones and histones [199, 200, 201, 202]. Several studies indicate that the acidic regions of chaperones can actually establish non‐electrostatic contacts with histones, thereby contributing to substrate specificity of histone chaperones [203, 204, 205].

The acidic disordered stretches of histone chaperones display a high prevalence of acidic amino acids but a low number of aromatic or hydrophobic residues [195]. They behave as IDRs since the electrostatic repulsion between the negatively charged side chains keeps them flexible and unstructured in solution [206]. IDRs exhibit a wide ensemble of conformational states [207]. Such suppleness allows the adoption of different conformations when binding to a protein partner—a phenomenon commonly known as ‘fuzziness’. Fuzziness adds flexibility, conformational heterogeneity and versatility to the protein–protein recognition processes, thus facilitating complex regulation [208]. IDRs can act as molecular hubs, showing multivalent interactions with multiple partners within the same stretch of amino acids [209]. The LCAR‐involving complexes are driven by a high number of transient contacts with fast association and dissociation rates. Interestingly, the acidic disordered stretches of histone chaperones take advantage of these features for binding histones, allowing a more precise and adaptive complex formation [195].

As discussed above, several histone chaperones are able to interact with C*c* [26, 27, 28]. More in‐depth studies of C*c* specifically complexed to SET/TAF‐Iβ and NRP1 showed that C*c* interferes with the nucleosome assembly activity of the two chaperones [31, 132]. Plasmid supercoiling and nucleosome assembly assays showed that C*c* complexed with chaperones impairs the function of the latter. C*c* competes with histones for binding to SET/TAF‐Iβ and NRP1, as inferred from 1D ^1^H nuclear magnetic resonance (NMR) and electrophoretic mobility shift assays [31, 132]. Furthermore, 2D [^1^H‐^15^N] NMR titration experiments revealed a spread pattern of residues on the C*c* surface affected by binding to histone chaperones, in particular residues at the haem‐surrounding area and the face opposite to the haem crevice [210, 211, 212, 213]. Such experiments suggest that C*c* forms fuzzy complexes with histone chaperones, as reported in other systems [208]. It is noteworthy that the haem‐centred surface area of C*c* is crucial for non‐redox interactions with histone chaperones, as this surface of *c*‐type cytochromes is, in general, for electron transfer in well‐known respiratory and even photosynthetic complexes [214, 215, 216, 217, 218, 219, 220, 221, 222].

C*c* and histones share some biophysical features, namely a molecular weight of *ca*. 12 kDa and a highly positive electrostatic surface potential [223, 224, 225]. Based on such common physicochemical properties and their direct competition for binding to histone chaperones, it is plausible that C*c* specifically targets the acidic regions of histone chaperones, as do histones, thus explaining how C*c* might alter histone eviction/deposition to facilitate the action of DNA repair factors (Fig. 1). Further experimental work is however required to make general the molecular recognition mechanisms of C*c* towards the acidic regions of chaperones by exploring other C*c*:chaperone complexes, such as those involving the ANP32 protein family members, NCL or hnRNP C1/C2 [27, 28].

## Nuclear condensates result from electrostatically driven LLPS: examples modulated by Lys‐rich proteins

Many nuclear processes—for example DNA transcription, DNA repair, RNA processing, pre‐ribosome assembly—occur within nuclear condensates that compartmentalize and concentrate the required protein and nucleic acid molecules [226]. Such nuclear condensates exhibit emergent properties and common features that provide the cell with particular regulatory capabilities [226]. In addition to the LLPS‐mediated DNA repair foci addressed in Role of chromatin modifiers in DNA foci and their regulation by nuclear cytochrome *c*, chromatin stands out as nuclear condensates where histone proteins and DNA can display liquid‐like features. We discuss below the role of histone lysines in regulating the formation of nuclear condensates.

### Role of lysines in LLPS

Cation–π and electrostatic interactions are the main driving forces of biomolecular condensation. In this context, Tyr and Arg residues have been identified to be essential in LLPS leading to condensates of FUS family proteins, while Gly residues regulate droplet fluidity and Gln and Ser residues promote droplet hardening [227]. Nucleophosmin (NPM) condensates at the granular component of the nucleolus are mediated by its binding to proteins bearing multivalent Arg‐rich motifs [228, 229]. Another example of Arg‐mediated LLPS is the condensates formed by heterogeneous nuclear ribonucleoprotein A2 (hnRNP A2), which possesses a charged residue‐rich LC domain that is crucial for LLPS because methylation of Arg residues by protein–arginine methyl transferase 1 (PRMT1) hampers phase transition by disrupting cation–π interactions between aromatic and Arg residues [230]. Similar studies have been performed with DEAD‐box helicase 4 (Ddx4) and FUS proteins [231, 232, 233].

**Fig. 2:**
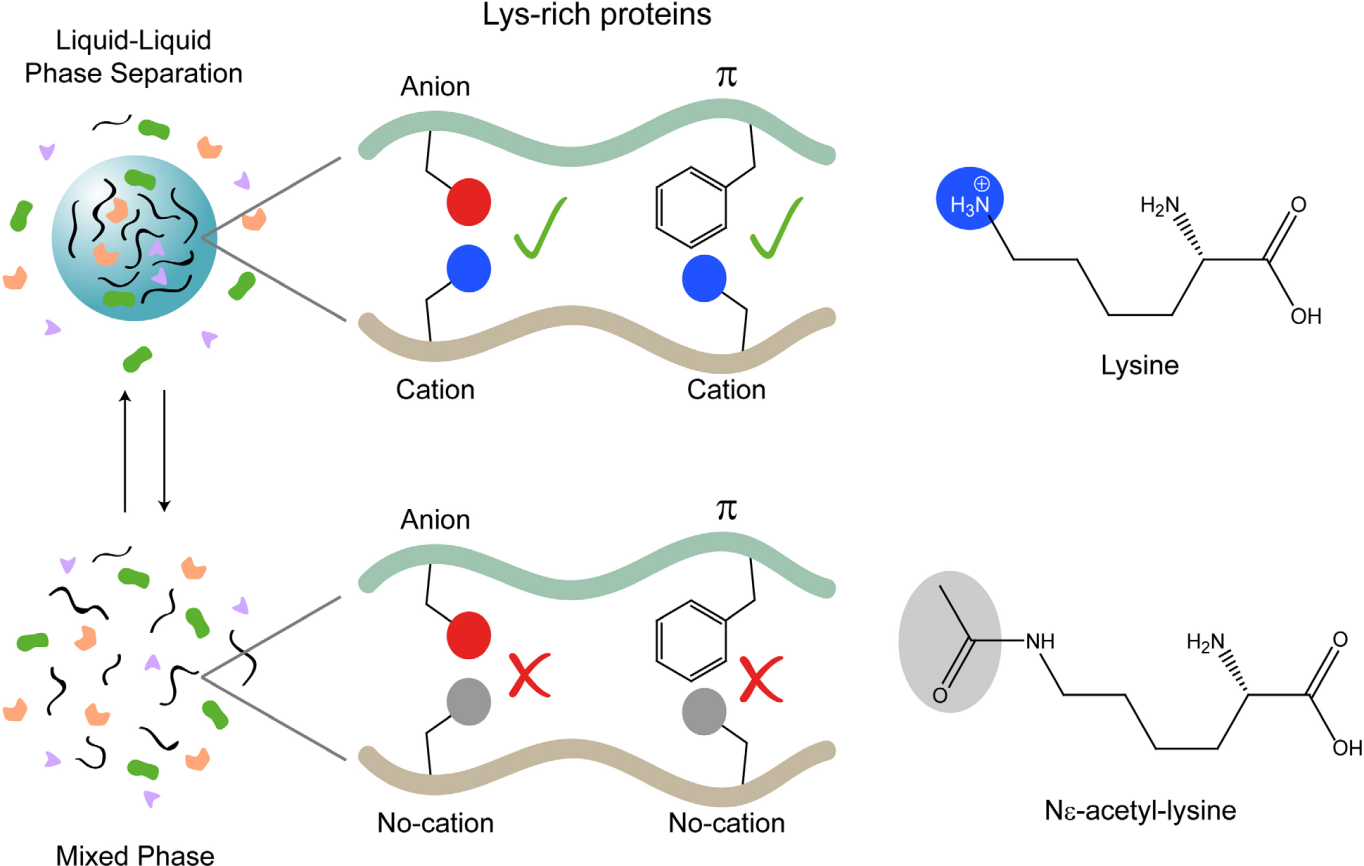
Nuclear condensate formation is dependent on PTMs of lysines. Positively charged residues of IDRs mediate LLPS. In fact, Lys residues in IDRs facilitate cation–anion and cation–π interactions within droplets formed by LLPS, whereas lysine acetylation impairs such interactions and, consequently, LLPS.

Like arginine, lysine can establish cation‐π and electrostatic contacts (Fig. 2). The Lys side chain is positively charged at physiological pH and is one of the most frequently post‐translationally modified amino acids [234]. Over the past few years, the spotlight was turned on the involvement of Arg residues in LLPS, but several recent studies have pointed out that Lys residues could also participate in biomolecular condensation and its regulation by PTMs [235, 236, 237]. A comparison between the physicochemical properties of Lys‐rich and Arg‐rich condensates revealed that Lys‐rich/RNA condensates are more dynamic and differ from the Arg‐rich/RNA coacervates, which are over 100 times more viscous [235, 237]. Both Arg and Lys residues have the same electrostatic charge at physiological pH, but the structure and geometry of their side chains modulate interaction with other molecules. The planar guanidinium group of arginine facilitates the cation–π interactions with aromatic residues and is involved in π–π contacts [238], in contrast with the weaker directional preference of the lysine ammonium group. The number and nature of hydrogen bonds formed by the ammonium and guanidinium cations differ as well. Lysine can actually form more hydrogen bonds than arginine, and the bond angle formed by lysine is distorted to 120° in contrast to the almost perfectly co‐linear bond formed by the guanidinium group [239, 240]. Such differences are proposed to weaken the interactions of Lys residues with RNA, thereby increasing the diffusion time of Lys‐rich *versus* Arg‐rich peptides in droplets [235, 237]. Altogether, these findings provide the fundamental principles to understand how droplet assembly, dynamics and multiphase coexistence are regulated by Arg/Lys residues.

### Histones as Lys‐rich proteins taking part in condensates

Recent studies have suggested that heterochromatin may possess liquid droplet‐like properties [241]. In this way, nuclear separation of silenced heterochromatin from actively transcribed euchromatin is in part driven by LLPS [242, 243]. This seems to be sufficient to produce the compaction degree necessary to organize the genome in the nucleus [244]. Since histones package cellular DNA into chromatin, it is not surprising that these proteins contribute to heterochromatin formation through reversible LLPS with DNA.

It has been reported that a mixture of the four core histones and the linker histone H1 undergoes LLPS with double‐stranded DNA [245]. Recently, it was also demonstrated that histone H1 condenses into liquid‐like droplets with DNA *in vitro* [245, 246], as well as with both DNA and nucleosomes in cell nuclei [247]. Such a H1‐mediated phase separation observed in nuclei is in agreement with the higher net positive charge and greater structural disorder of H1 compared with core histones [247]. Regarding the core histones, only H2A was able to induce droplet formation in the presence of DNA and nucleosomes, but to a lesser extent than H1 [247]. Interestingly, the other core histones (H2B, H3 and H4) precipitated under identical conditions. These studies strongly support a key role for histones in LLPS‐mediated formation of heterochromatin domains [247].

Histone tails drive the formation of liquid condensates as they behave as IDRs involved in weak and often reversible interactions with several ligands and neighbouring nucleosomes [244, 248]. Mutation of Lys and Arg residues in the histone H4 tail leads to a chromatin defective in droplet formation, thus revealing the vital role of contacts between positively charged histone tails and negatively charged DNA molecules in chromatin LLPS [244].

Linker histone H1, DNA lengths between nucleosomes, histone PTMs and nuclear proteins exhibiting phase separation properties might regulate chromatin LLPS, thereby contributing to chromatin reorganization and compartmentalization [244, 248, 249]. All these factors also finetune droplet properties to form condensates of different density, similar to the behaviour of chromatin inside cells [244, 249].

### Effect of lysine acetylation on nuclear condensates

Lys residues undergo PTMs, including acetylation, methylation, ubiquitylation, SUMOylation and glycation, among others [250]. In particular, lysine acetylation leads to neutralization of its positive electrostatic charge and thereby impairs its cation–anion and cation–π interactions (Fig. 2). With this in mind, Matthias and co‐workers showed that deacetylation of DEAD box RNA helicase 3 X‐linked (DDX3X) is necessary for robust LLPS and, consequently, for stress granule maturation [236].

**Fig. 3:**
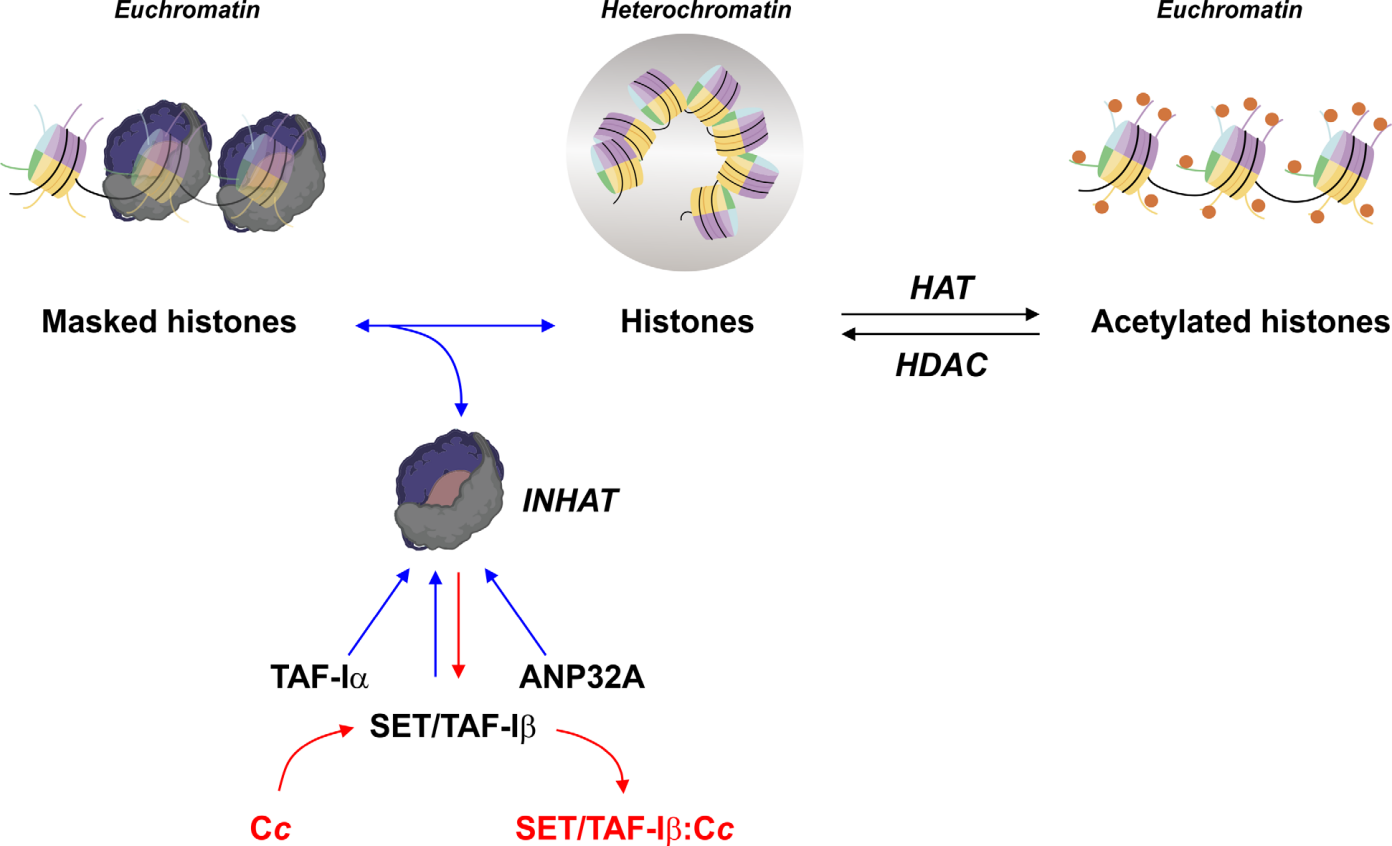
Interplay among HAT, INHAT and C*c* in chromatin remodelling. Enzymatic acetylation and deacetylation of lysines at the N‐terminal tail of histones, mainly H3 and H4, are responsible for chromatin remodelling, thereby regulating the reversible condensation of euchromatin into heterochromatin, with a more compacted structure. HAT catalyses the acetyl group transfer for lysine acetylation, whereas HDAC plays the antagonistic role. The action of HAT can, however, be inhibited by the INHAT complex, which binds to deacetylated histones to cause a ‘masking effect’ that decreases their net positive charge, which is essential for histone–histone and histone–DNA electrostatic interactions. As the INHAT complex is composed of TAF‐Iα, SET/TAF‐Iβ and ANP32A proteins, the action of INHAT can in turn be impaired by C*c*, which binds to SET/TAF‐Iβ upon DNA break and translocation of C*c* into the nucleus.

The dynamics of chromatin condensation/decondensation are essential for several cell processes, including gene regulation, the DDR and cell differentiation [251, 252, 253]. Histones undergo different PTMs that alter their interaction with DNA and other histones (Table 1) [254, 255, 256, 257]. In particular, acetylation is a reversible PTM that introduces an acetyl group from acetyl‐CoA into the ɛ‐amino group of lysine. Specifically, acetylation of Lys residues in histone N‐terminal tails—mainly H3 and H4—is related to chromatin decondensation (or formation of euchromatin), by neutralizing lysine positive charges and enabling specific electrostatic interactions between histones and DNA [258, 259, 260], whereas the absence of lysine modifications allows chromatin condensation (or formation of heterochromatin) by reversible LLPS (Fig. 3). Recently, Rosen and co‐workers described the condensation and LLPS of acetylated chromatin [244] with histone acetylated lysines acting as binding platforms for bromodomain‐containing proteins (bromodomains) involved in gene transcription and chromatin remodelling [261, 262]. Although bromodomains allow acetyl‐chromatin condensation, the resulting droplets have singular physicochemical properties and are non‐miscible with unmodified chromatin droplets [244].

**Table 1:** Post‐translationally modifications and position numbers of histone lysines.

Histone	Acetylation (ac)	Methylation (me)	Biotinylation (bio)	Crotonylation (cr)	Propionylation (prop)	Phosphorylation (ph)	Butyrylation (buty)	Ubiquitination (ub)	Sumoylation (su)	ADP rybosylation (ar)
H2A	5		9					119		13
H2B	5,12,15, 20	120		5		14				30
H3	4, 9, 14^a^, 23^a^, 27, 36^a^	4, 9, 27, 36, 79			23					27, 37
H4	5^a^, 8^a^, 12, 16^a^, 20^a^,91	20					5	91	14	16
H2AX	5							119		

^a^ Recognized by bromodomain.

As mentioned in SET/template‐activating factor‐Iβ, histone acetylation and deacetylation reactions are catalysed by HATs and HDACs, respectively [107, 108] (Fig. 3). Histone acetylation not only plays a crucial role in transcriptional upregulation [109, 263, 264] but is also required for recruitment of the DDR effector proteins [265]. In fact, growing evidence supports a role for histone acetylation in DNA repair [72]. Thus, residue acetylation at a specific position followed by deacetylation is relevant for viability after DNA repair during homologous recombination [266], which suggests that dynamic changes in histone acetylation accompany DSB repair. The pattern of acetylation is highly conserved among eukaryotes, highlighting the importance of this PTM in chromatin remodelling [267]. As also mentioned in SET/template‐activating factor‐Iβ, another regulatory mechanism is based on inhibition of the HAT activity of p300/CBP and PCAF acetyltransferases exerted by the INHAT complex, which impairs lysine acetylation by binding to histones (Fig. 3) [105, 160, 268]. Thus, the INHAT complex inhibits histone acetylation through a ‘histone masking’ mechanism, which consists in hindering the histone surface from acetyltransferases [105] (Fig. 3).

SET/TAF‐Iβ, as a component of the INHAT complex, mediates nucleosome assembly and acts in the DDR by preventing the binding of several chromatin modulator factors to DNA, thereby resulting in DNA condensation [91, 121]. Under DNA damage, C*c* migrates from mitochondria to the nucleus, where it interacts with SET/TAF‐Iβ and impairs its nucleosome assembly activity [31]. The degree of such inhibition can be regulated by the amount of C*c* that reaches the nucleus [130]. On the basis of these findings, nuclear C*c* emerges as an additional regulating agent of histone acetylation by blocking SET/TAF‐Iβ, and, consequently, INHAT complex functionality (Fig. 3).

DNA methylation—which is another hallmark of chromatin condensation—and histone acetylation depend on one another, thus resulting in crosstalk mediated by SET/TAF‐Iβ as DNA demethylation inhibition is also mediated by the histone chaperone [269, 270]. A fine balance between DNA methylation and histone modification at the level of lysines thus has significant implications for understanding cell development, reprogramming and tumorigenesis [108, 271].

## Conclusions and perspectives

In addition to its well‐established functions in mitochondrial metabolism and apoptosis, growing evidence reveals an astounding, unexpected role for C*c* in the cell nucleus upon DNA damage. In the nucleus, this hemeprotein binds to several histone chaperones involved in chromatin remodelling following the DDR. Since nuclear C*c* interferes with the nucleosome assembly activities of such chromatin factors, it might likewise alter chromatin dynamics after DNA insults. The role of C*c* in the nucleus may actually be wider if the hemeprotein regulates INHAT and/or PP2A activities by binding to histone chaperones, for example SET/TAF‐Iβ. In this way, nuclear C*c* emerges not only as a major regulatory agent in DNA repair through its fine‐tuning of nucleosome assembly activity and, likely, nuclear condensate formation, but also moonlights as a key master protein of cell life and death.

## Conflict of interest

The authors declare no conflict of interest.
